# Predicting the Need for Desmopressin Treatment During Inpatient and After Discharge Following Endoscopic Sellar Surgery

**DOI:** 10.3389/fneur.2022.843646

**Published:** 2022-02-17

**Authors:** Chia-En Wong, Wei-Hsin Wang, Ming-Ying Lan, Po-Hsuan Lee, Chi-Chen Huang, Pei-Fang Su, Jung-Shun Lee

**Affiliations:** ^1^Section of Neurosurgery, Department of Surgery, College of Medicine, National Cheng Kung University Hospital, National Cheng Kung University, Tainan, Taiwan; ^2^Department of Neurosurgery, Taipei Veterans General Hospital, Taipei, Taiwan; ^3^School of Medicine, National Yang Ming Chiao Tung University, Hsinchu, Taiwan; ^4^Department of Otolaryngology, Taipei Veterans General Hospital, Taipei, Taiwan; ^5^Department of Statistics, National Cheng Kung University, Tainan, Taiwan; ^6^Department of Cell Biology and Anatomy, College of Medicine, National Cheng Kung University, Tainan, Taiwan; ^7^Institute of Basic Medical Sciences, College of Medicine, National Cheng Kung University, Tainan, Taiwan

**Keywords:** postoperative diabetes insipidus, desmopressin (DDAVP), endoscopic endonasal approach (EEA), pituitary tumor, diabetes insipidus

## Abstract

**Introduction:**

Postoperative diabetes insipidus (DI) is a common complication following endoscopic sellar surgery. However, the requirement of desmopressin treatment for patients with DI are heterogenous. Although the predictors of postoperative DI have been reported, whether these patients required desmopressin treatment remained uninvestigated. Predicting the need of desmopressin can benefit clinical decision making more directly than predicting the occurence of postoperative DI. This study aimed to identify variables that predict the need for desmopressin treatment following sellar surgery.

**Methods:**

Patients undergoing endoscopic sellar surgery between 2016 and 2019 were retrospectively reviewed. Twenty-three variables, characterized as potential predictors for requiring desmopressin treatment, were analyzed. To assess the capability to generalize the identified predictors, external validation with receiver operating characteristic (ROC) analysis was performed using a second series from 2019 to 2020.

**Results:**

Postoperative DI occurred in 40 of 159 included patients. Twelve patients required inpatient desmopressin treatment and 20 patients needed desmopressin prescription after discharge. The potential predictors of requiring any desmopressin use included higher peak sodium (Na) level (*p* = 0.007), lower minimum Na level (*p* = 0.043), and higher peak urine output (*p* = 0.006), but these were not supported by external validation. The predictors of requiring desmopressin after discharge included higher peak Na (*p* = 0.040) and minimum Na levels (*p* = 0.048), which were supported by external ROC validation showing areas under curve of 0.787, 0.611, and 0.898 for peak Na (*p* = 0.036), minimum Na (*p* = 0.460), and peak Na – minimum Na levels (*p* < 0.001), respectively. A criterion of peak Na ≥ 150 mmol/L or peak Na – minimum Na ≥ 10 predicted the need of desmopressin prescription after discharge. A postoperative management algorithm was proposed.

**Conclusion:**

The required treatments for patients with postoperative DI following endoscopic sellar surgery are heterogenous. Elevated peak Na and large peak Na—minimum Na levels in the perioperative period predicted requiring desmopressin after hospital discharge. Patients with peak Na <150 mmol/L and peak Na—minimum Na <10 can be safely discharged without desmopressin prescription.

## Introduction

Postoperative diabetes insipidus (DI) following sellar surgery is a significant complication caused by intraoperative manipulation and interruption of the pituitary stalk (1–5). The degree of damage correlates with the severity of vasopressin deficits and produces a spectrum of water and electrolyte imbalance (6). Although the incidence rate of permanent DI has decreased in modern practice, transient postoperative DI remains common (2, 7, 8). In some patients, the duration of transient postoperative DI can last for weeks to months and necessitate desmopressin replacement (5, 7). Timely initiation of desmopressin treatment in patients presenting overt DI symptoms can prevent aggravation of electrolyte imbalance (5). The management of postoperative DI has been described, wherein a commonly accepted indication for desmopressin administration can be summarized as follows: (1) polyuria with urine output (UOP) > 300 mL/h for 2–3 h with low urine specific gravity (SPGR <1.005), (2) elevated serum sodium (Na) > 145 mEq/L, and (3) thirst and/or hypernatremia not alleviated by drinking water or patient intolerable to polyuria/polydipsia (5, 9, 10).

Nonetheless, not all patients with postoperative DI require desmopressin. Most transient DI has a typical duration of 3–5 days and gradually resolves over time (5, 11). A low threshold of desmopressin administration in such patients may inadvertently precipitate electrolyte imbalance and prolong hospitalization (1, 4). Moreover, timely discontinuation of desmopressin in patients with biphasic or triphasic DI responses is crucial to prevent syndrome of inappropriate secretion of antidiuretic hormone and hyponatremia (12). Clinicians must be vigilant about sudden electrolyte fluctuations and knowledgeable in the course of postoperative DI to avoid potential complications. Although the predictors of postoperative DI have been reported and there is evidence on the treatment of postoperative DI (2, 3, 7, 13, 14), whether a patient with DI may require desmopressin therapy and the strategy for desmopressin discontinuation are not yet investigated. Determining an optimal indication and duration of desmopressin treatment may improve patient care and prevent complications associated with suboptimal treatment duration, such as rebounding of DI symptoms and iatrogenic hyponatremia (15). This will be especially helpful for determining whether desmopressin can be discontinued prior to discharge patients.

This study aimed to analyze the risk factors of requiring desmopressin treatment following sellar surgery by exploring the differences in DI manifestations among patients treated with and without desmopressin. We also provided the early postoperative management strategy in our practice, including fluid management, laboratory testing, diagnosis, treatment, and discharge plan.

## Materials and Methods

The present study was a retrospective two-center analysis of patients who underwent endonasal endoscopic approach (EEA) sellar surgeries. This study was approved by the institutional review board of both institutions (NCKU-IRB-Approval No. A-ER-110-147/VGHTPE-IRB-Approval No. 2021-06-011AC). Considering the retrospective design of this study, informed consent was not required.

Patients undergoing EEA from January 2016 to April 2021 were included. The patients were divided into a cohort from January 2016 to December 2019 (Cohort 2016–2019) to identify the potential risk factors for requiring desmopressin and a validation cohort from January 2020 to April 2021 (Cohort 2020–2021) to externally validate the predictors. Both cohorts were retrospectively reviewed and predictors identified in Cohort 2016–19 were not used to prospectively guide the management in Cohort 2020–21. We excluded patients with preoperative DI and undergoing microscopic, transcranial, or combined surgery. Patients who previously underwent non-endoscopic surgeries were included if they subsequently underwent endoscopic surgeries. A duration of at least 6 months was determined as an adequate follow-up. Data for all patients were compiled from electronic medical records and clinical notes. We reviewed their radiological reports to analyze the tumor size and suprasellar extension (16–19).

The patients were considered to have DI if it was documented in their daily progress, discharge notes, or clinic notes. The criteria to diagnose DI required all of the following: polyuria with UOP > 300 mL/h for 2–3 consecutive hours, low urine SPGR <1.005, and serum Na > 145 mEq/L (8, 9, 20, 21). In the postoperative period, no fluid restrictions were made, but patients were instructed to drink only when thirsty to prevent overhydration. UOP and SPGR were monitored every 2–4 h for at least the first 24 h. Serum Na levels were measured if the patients' UOP and SPGR met the criteria. If patients diagnosed with DI could not tolerate the DI symptoms or could not maintain fluid balance by drinking water, single-dose desmopressin was given (5, 9, 10). In our practice, patients were monitored for at least 4 postoperative days (PODs) to ensure full resolution of transient DI or to identify prolonged DI. During this period, the measured peak Na value, minimum Na value, and the difference between the first postoperative and the last preoperative Na (ΔNa) were documented. A visit by a rhinologist was usually arranged at POD 4 to remove nasal packing and check for early postoperative cerebrospinal fluid leak. Uncomplicated patients were discharged on PODs 4–6. The duration of DI was calculated from the first time the patient met the criteria of DI diagnosis to the time of desmopressin discontinuation and resolution of DI symptoms with normal UOP and serum Na levels. Permanent DI was defined as > 6 months.

Statistical analysis for categorical variables was performed using the χ^2^ test or Fisher's exact test. Nonparametric variables were compared using the Mann–Whitney *U* test. Univariate logistic regression was performed to identify potential predictors. Multivariate logistic regression models were constructed using variables with *p* ≤ 0.2 in the univariable models. We performed receiver operating characteristic (ROC) analyses in both Cohort 2016–2019 and Cohort 2020–2021 for internal and external validations, respectively. Cut-off values were determined using the Youden index. Statistical tests were conducted using MedCalc 19.7.2 (MedCalc Software Ltd.). A *p* <.05 was considered statistically significant.

## Results

A total of 159 patients were included, with 92 patients from 2016 to 2019 and 67 patients from 2020 to 2021. In the cohort from 2016–2019, the median age was 55 (range, 22–83) years, and 70.7% were female. The median follow-up duration was 1,021 (range, 725–1,423) days. The overall incidence rate of postoperative DI was 26.1% (24/92), including 21.7% (20/92) and 4.3% (4/92) of transient DI and permanent DI, respectively. In the validation cohort (2020–2021), the median age was 54 (range, 20–84) years, and 55.2% were female. The median follow-up duration was 414 (range, 185–634) days. The incidence rates of transient and permanent DI were 19.4 and 3.0%, respectively.

The clinical and outcomes metrics based on whether desmopressin was required are shown in Table 1. Among the 92 patients, 21 were treated with desmopressin, including 14 patients requiring desmopressin prescription after discharged from hospital. In these patients, 17 and 4 had transient and permanent DI, respectively. Four patients having transient DI did not require desmopressin and had a shorter DI duration (*p* = 0.015). The length of hospitalization (LOH) was longer in patients requiring desmopressin than in those not requiring desmopressin (*p* < 0.001). Requiring desmopressin was associated with a higher peak Na level (*p* < 0.001), a lower minimum Na level (*p* = 0.004), and a larger peak Na—minimum Na difference (*p* < 0.001) measured in the perioperative period from the last preoperative value to POD 4. Patients requiring desmopressin had higher peak UOP (*p* < 0.001), greater superior-inferior tumor diameter (*p* = 0.023), and increased percentage of craniopharyngioma, Rathke cleft cyst (RCC), chordoma, and pituicytoma (*p* = 0.011).

**Table 1:** Demographics and outcomes in patients undergoing EEA for sellar pathologies.

**Patients**	**Cohort 2016–19**			**Cohort 2020–21**			***p*-value**
**Characteristics**	**Desmopressin usage**		***p*-value**	**Desmopressin usage**		***p*-value**	
	**No (*n* = 71)**	**Required (*n* = 21)**		**No (*n* = 56)**	**Required (*n* = 11)**		
**Outcomes**							
No DI	67 (94.4)	0	**<0.001**	52 (92.9)	0	**<0.001**	0.834
Transient DI	4 (5.6)	17 (81.0)		4 (7.1)	9 (81.8)		
Permanent DI	0	4 (19.0)		0	2 (18.2)		
DI duration (days)	3 (2–3)^*^	21 (9–142)	**0.015**	3.5 (3–4)^**^	9 (4–143.5)	0.184	0.069
LOH (days)	7 (6–8)	11 (8–16)	**<0.001**	6.5 (6–7.5)	8 (7–10.5)	**0.003**	0.187
Duration of desmopressin							
Inpatient only		7 (33.3)			5 (45.5)		
Required after discharge		14 (66.7)			6 (54.5)		
ER visit or readmission	6 (8.5)	5 (23.8)	0.058	9 (16.1)	2 (18.2)	0.864	0.423
CSF leak	15 (21.1)	1 (4.8)	0.084	8 (14.3)	2 (18.2)	0.742	0.679
Post-op SIADH	5 (7.0)	0	0.214	5 (8.9)	0	0.307	0.604
**Demographics**							
Age (years)	57 (43–63)	53 (41.5–63)	0.642	56 (39–66)	49 (42.5–54.5)	0.229	0.516
Sex: Female	51 (71.8)	14 (66.7)	0.650	31 (55.4)	6 (54.5)	0.961	0.055
Pre-op hormone disturbance	21 (29.6)	2 (9.5)	0.064	18 (32.1)	2 (18.2)	0.359	0.498
Pre-op visual disturbance	35 (49.3)	12 (57.1)	0.530	29 (51.8)	8 (72.7)	0.205	0.607
Prior surgery	12 (16.9)	5 (23.8)	0.476	8 (14.3)	1 (9.1)	0.647	0.397
Apoplexy	6 (8.5)	3 (14.3)	0.432	9 (16.1)	0	0.156	0.475
**Sodium measurements (pre-op–POD4)**						
Peak Na level (mEq/L)	143 (142–145)	149.5 (146–155)	**<0.001**	144 (142–145.5)	148 (145.5–151)	**0.001**	0.413
Minimum Na level (mEq/L)	139 (137–141)	137 (135.5–139)	**0.004**	139.5 (138–141)	139 (137–141)	0.614	**0.041**
Peak Na–Minimum Na (mEq/L)	4 (3–6)	11 (9–15)	**<0.001**	4 (3–6)	9 (8–12)	**<0.001**	0.122
ΔNa level (mEq/L)	1 (−1–3)	1 (-2–4)	0.674	0 (-1–2)	1 (-1–4)	0.615	0.811
**Daily UOP**							
Peak UOP prior to desmopressin (ml/day)	3,510 (2,835–4,065)	5,650 (5,035–7,645)	**<0.001**	3,300 (2,820–4,105)	6,110 (5,560–6,600)	**<0.001**	0.779
Time of peak UOP							
POD 0	24 (33.8)	4 (19.0)	0.186	16 (26.8)	4 (36.4)	0.117	0.963
POD 1	28 (39.4)	7 (33.3)		21 (37.5)	3 (27.3)		
POD 2	11 (15.5)	3 (14.3)		11 (19.6)	3 (27.3)		
POD >2	8 (11.3)	7 (33.3)		8 (14.3)	1 (9.1)		
**Tumor size**							
SI diameter (cm)	1.95 (1.37–2.73)	2.30 (1.91–3.54)	**0.023**	2.38 (1.72–2.96)	1.91 (1.66–2.27)	0.194	0.420
AP diameter (cm)	1.84 (1.31–2.35)	2.02 (1.52–2.70)	0.252	1.78 (1.48–2.27)	1.86 (1.28–2.21)	0.877	0.155
LR diameter (cm)	2.27 (1.67–3.09)	2.35 (2.08–2.88)	0.322	2.33 (1.76–2.91)	2.04 (1.75–2.94)	0.530	0.233
Suprasellar extension	36 (50.7)	12 (57.1)	0.369	37 (66.1)	5 (45.5)	0.200	0.237
**Pathology**							
Pituitary adenoma	53 (74.6)	11 (52.4)	**0.011**	47 (83.9)	4 (36.4)	**0.003**	0.880
Rathke's cleft cyst	6 (8.5)	2 (9.5)		2 (3.6)	2 (18.2)		
Meningioma	7 (9.9)	1 (4.8)		3 (5.4)	1 (9.1)		
Craniopharyngioma	1 (1.4)	4 (19.0)		1 (1.8)	3 (27.3)		
Chordoma	1 (1.4)	2 (9.5)		1 (1.8)	0		
Metastasis	2 (2.8)	0		1 (1.8)	0		
Pituicytoma	0	1 (4.8)		0	1 (9.1		
Epidermoid cyst	1 (1.4)	0		1 (1.8)	0		

*Results were reported as median with IQR or number with percentage*.

* *In 4 patients in Cohort 2016–19 with transient DI who received no desmopressin*.

** *In 4 patients in Cohort 2020–21 with transient DI who received no desmopressin*.

*Bold values indicate p-value < 0.05*.

**Figure 1:**
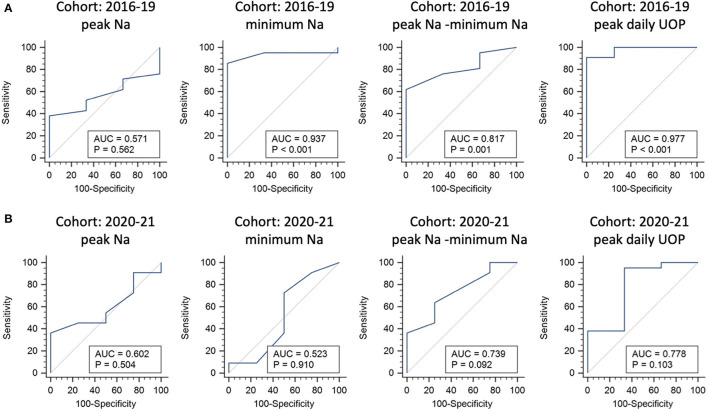
Internal and external validations for predictors of requiring any desmopressin treatment following endonasal endoscopic approach. Receiver operating characteristic curve analysis using the 2016–2019 cohort for internal validation **(A)** and 2020–2021 cohort for external validation **(B)**.

Multivariate regression analysis identified the potential predictors of requiring any desmopressin treatment, including greater peak Na level (*p* = 0.007), lower minimum Na level (*p* = 0.043), and greater peak UOP (*p* = 0.006) (Table 2). ROC analysis showed the areas under curve (AUCs) were 0.571, 0.937, 0.817, and 0.977 for peak Na level (*p* = 0.562), minimum Na level (*p* < 0.001), peak Na—minimum Na level (*p* = 0.001), and peak UOP (*p* < 0.001), respectively (Figure 1A). External validation using the validation cohort (Cohort 2020–2021, Table 1) showed the AUCs were 0.602, 0.523, 0.739, and 0.778 for peak Na level (*p* = 0.504), minimum Na level (*p* = 0.910), peak Na—minimum Na level (*p* = 0.092), and peak UOP (*p* = 0.103), respectively (Figure 1B).

**Table 2:** Logistic regression model of requiring any desmopressin use in patients with postoperative DI following EEA.

**Characteristics**	**Univariate logistic regression**			**Multivariate logistic regression**		
	**OR**	**95% CI**	***p*-value**	**OR**	**95% CI**	***p*-value**
**Demographics**						
Age (years)	0.995	0.962–1.029	0.767			
Female	0.784	0.275–2.227	0.648			
Pre-op hormone disturbance	0.251	0.054–1.173	0.079			
Pre-op visual disturbance	1.371	0.514–7.660	0.528			
**Sodium measurements (pre-op–POD4)**			
Peak Na level (mEq/L)	1.682	1.324–2.137	**<0.001**	1.953	1.207–3.159	**0.007**
Minimum Na level (mEq/L)	0.796	0.661–0.958	**0.016**	0.523	0.280–0.979	**0.043**
ΔNa level (mEq/L)	1.018	0.874–1.185	0.824			
**Daily UOP**						
Peak UOP	1.001	1.001–1.002	**<0.001**	1.002	1.001–1.003	**0.006**
Time of peak UOP	1.526	1.007–2.312	**0.047**	1.536	0.492–4.498	0.460
**Tumor size**						
SI diameter	1.949	1.161–3.274	**0.012**	0.958	0.190–24.845	0.960

*Bold values indicate p-value < 0.05*.

**Figure 2:**
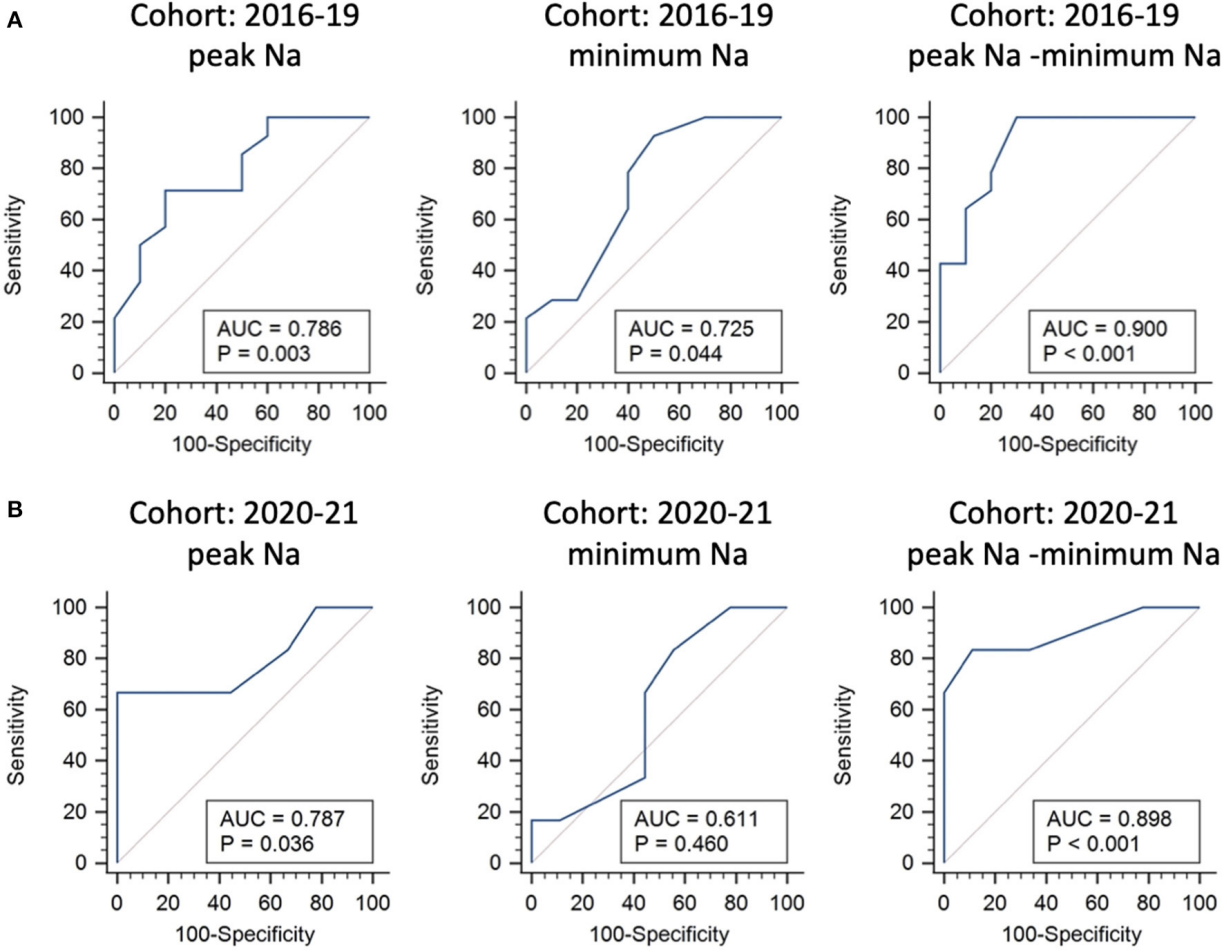
Internal and external validations for predictors of requiring desmopressin prescription after hospital discharge following endonasal endoscopic approach. Receiver operating characteristic curve analysis using the 2016–2019 cohort for internal validation **(A)** and 2020–2021 cohort for external validation **(B)**.

**Figure 3:**
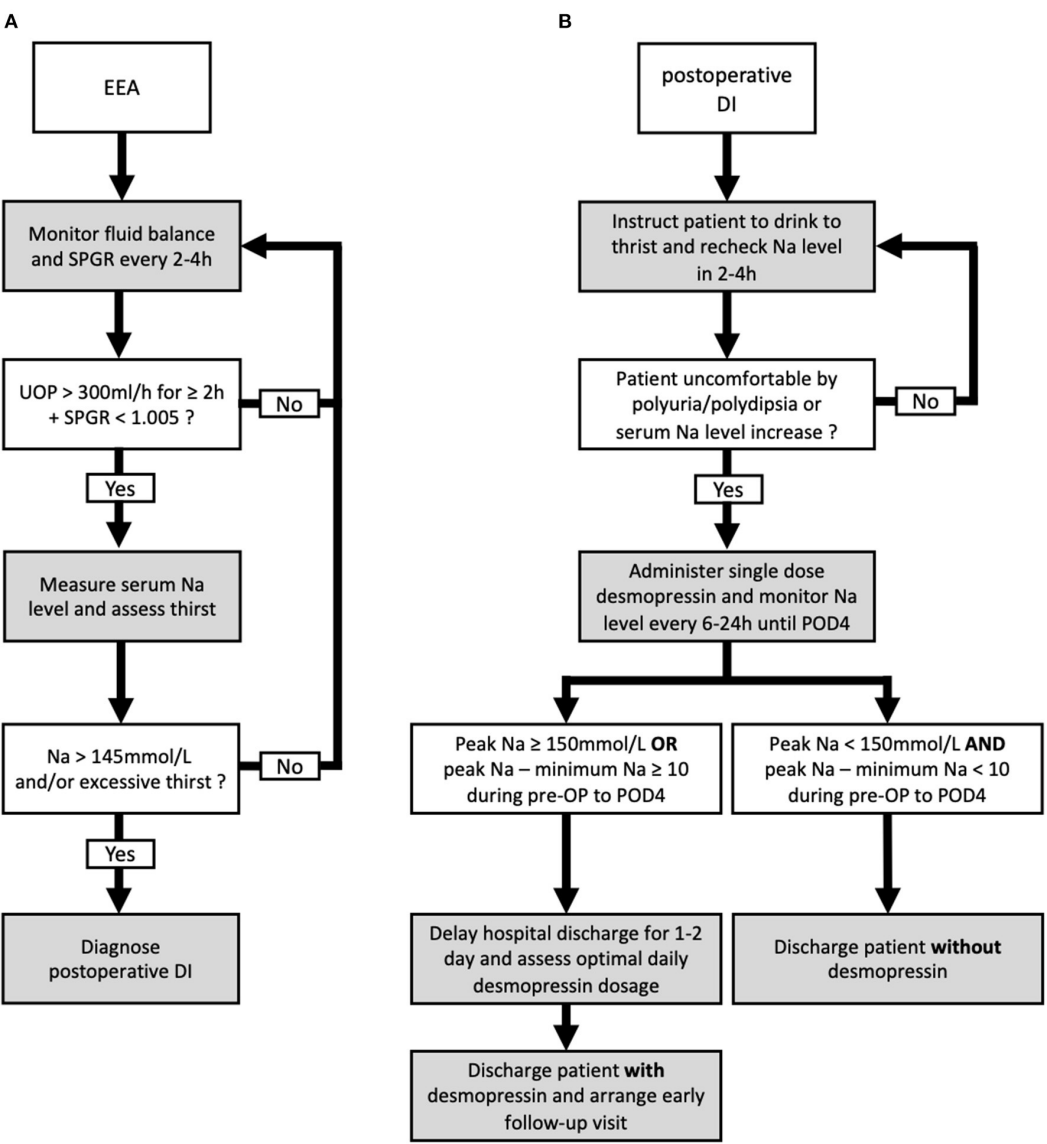
Proposed algorithm for the diagnosis and management of postoperative diabetes insipidus (DI) following endonasal endoscopic approach (EEA). The proposed diagnostic **(A)** and management **(B)** algorithms including the discharge plan for postoperative DI following EEA.

The potential predictors of requiring desmopressin prescription after hospital discharge included greater peak Na level (*p* = 0.040) and lower minimum Na level (*p* = 0.048) (Table 3). ROC analysis showed that the AUCs were 0.786, 0.725, and 0.9 for peak Na level (*p* = 0.004), minimum Na level (*p* = 0.044), and peak Na—minimum Na level (*p* < 0.001), respectively (Figure 2A). External validation with the validation cohort showed that the AUCs were 0.787, 0.611, and 0.898 for peak Na level (*p* = 0.036), minimum Na level (*p* = 0.460), and peak Na—minimum Na level (*p* < 0.001), respectively (Figure 2B). The cut-off values, sensitivities, specificities, and predictive ratios are shown in Table 4. Based on the identified and validated predictors, we proposed the early postoperative strategies for the diagnosis and management of postoperative DI in Figure 3.

**Table 3:** Logistic regression model of requiring desmopressin prescription at discharge.

**Characteristics**	**Univariate logistic regression**			**Multivariate logistic regression**		
	**OR**	**95% CI**	***p*-value**	**OR**	**95% CI**	***p*-value**
**Demographics**						
Age (years)	0.986	0.948–1.026	0.488			
Female	0.707	0.213–2.165	0.571			
Pre-op hormone disturbance	0.452	0.095–2.192	0.325			
Pre-op visual disturbance	1.895	0.582–6.165	0.288			
**Sodium measurements (pre-op–POD4)**			
Peak Na level (mEq/L)	1.303	1.014–1.674	**0.039**	1.854	1.029–3.337	**0.040**
Minimum Na level (mEq/L)	0.650	0.418–1.012	0.057	0.341	0.117–0.991	**0.048**
ΔNa level (mEq/L)	0.976	0.798–1.194	0.812			
**Daily UOP**						
Peak UOP	1.000	0.999–1.001	0.727			
Time of peak UOP	1.644	0.778–3.473	0.193	1.517	0.366–6.287	0.566
**Tumor size**						
SI diameter	1.629	0.691–3.840	0.265	1.168	0.199–6.859	0.864

*Bold values indicate p-value < 0.05*.

**Table 4:** The performance of predictors of requiring desmopressin prescription at discharge.

**Predictors**	**AUC**	***p*-value**	**cut-off**	**Sensitivity**	**Specificity**	**PPV**	**NPV**
**Internal validation (Cohort 2016–19)**				
Peak Na level (mEq/L)	0.786	**0.003**	≥ 150	71.4	80.0	83.3	66.7
Minimum Na level (mEq/L)	0.725	**0.044**	≤ 139	92.9	50.0	72.2	83.8
Peak Na–minimum Na level (mEq/L)	0.900	**<0.001**	≥ 10	100.0	70.0	82.5	100.0
**External validation (Cohort 2020–21)**		
Peak Na level (mEq/L)	0.787	**0.036**	≥ 150	66.7	100.0	100	81.8
Minimum Na level (mEq/L)	0.611	0.460	≤ 140	83.3	44.4	50.0	80.0
Peak Na–minimum Na level (mEq/L)	0.898	**<0.001**	≥ 10	83.3	88.9	83.3	88.9

*Bold values indicate p-value < 0.05*.

## Discussion

Desmopressin is commonly used to treat postoperative DI. In the present study, 32 out of 40 patients with DI required at least a single dose of desmopressin, in which 20 required desmopressin after hospital discharge. Our results showed patients requiring desmopressin treatment were associated with longer LOH, higher UOP, greater perioperative Na fluctuation, and larger cranio-caudal tumor size. External validation showed peak Na and peak Na-minimum Na levels were predictors of requiring desmopressin after hospital discharge.

Postoperative DI in our study was most often transient, which occurred in 21.4 % of patients, whereas permanent DI occurred in only 3.8% of patients. Previous studies have reported the incidence rates of postoperative DI ranging from 8 to 30% and up to 40 to 60% for pituitary adenomas and RCC or craniopharyngiomas, respectively (3, 14, 22–25). Similarly, we found that patients with craniopharyngioma and RCC had a higher incidence rate of DI. This can be attributed to the proximity of these tumors' origins to the infundibulum, and intraoperative manipulation and damage are inevitable in gross total resection (8, 26, 27). Apart from the incidence of postoperative DI, we investigated the treatment required in these patients. Although the incidence rates and risk factors of DI occurrence were reported in previous publications, the percentage of patients requiring inpatient or prolonged desmopressin treatment was rarely reported and the descriptions of the desmopressin management strategies were unclear (13, 14, 22–27). In the present study, we highlighted that the clinical course and required treatment for patients with transient DI were heterogenic. Although 8 patients with transient DI were treated without desmopressin, 12 received inpatient desmopressin, and 14 required desmopressin prescription after discharge. Since not all patient with postoperative DI required desmopressin treatment and the treatment duration varied, identifying high-risk patients who require inpatient or after-discharge desmopressin treatment may be more beneficial to clinical practice than identifying patients who developed DI. Therefore, in the present analysis, we stratified the patients according to the duration of the required desmopressin treatment.

Schreckinger et al. used the difference of the first postoperative and the last preoperative Na (ΔNa) as a predictor of postoperative DI (9). Nonetheless, the difference of ΔNa among patient treated with or without desmopressin was not evident in our study. Immediate postoperative fluid and electrolyte imbalance in patients receiving EEA is common, which can be attributed to perioperative fluid administration, water and Na retention caused by steroid administration, and increased sympathetic response as an adaptation to stressors (1, 4, 28, 29). In contrast, electrolyte fluctuations caused by true vasopressin deficit may appear in later days after the initial postoperative diuresis phase, and this was also supported by our result showing a trend of delayed occurrence of peak UOP in patients with permanent DI. Therefore, measuring the Na values in the first few PODs may have a better chance to detect the electrolyte fluctuation caused by true vasopressin deficit. In a study by Sigounas et al., at least one occurrence of elevated serum Na > 145 within the first 5 PODs was used as a rule to identify patients at increased risk of DI in 120 endoscopic pituitary surgeries (7). However, measuring only the peak Na value may be subject to the variations in preoperative baseline Na level; hence, we also reported the minimum Na level in the perioperative period in this study.

Our results showed that although a lower minimum Na level and a greater peak Na—minimum Na level predicted requiring at least a single dose of desmopressin in internal validation (Cohort 2016–2019), both predictors were not supported by external validation using a second cohort (Cohort 2020–2021). It remained difficult to predict the need of incidental single-dose desmopressin in patients with DI. Although commonly accepted criteria of UOP, SPGR, and serum Na were proposed (5, 9, 10), the decision of desmopressin administration largely depends on whether the patients' symptoms were alleviated by drinking water. The variation in the individual's tolerance to large amounts of drinking and polyuria might largely interfere the decision to administer single-dose desmopressin during admission.

In contrast, the prediction of requiring desmopressin prescription at discharge using a greater peak Na and a greater peak Na—minimum Na level was validated in both internal and external validation cohorts. It is reasonable that the prediction of requiring desmopressin prescription at discharge is less interfered by the individual's tolerance. Since it would not be possible for patients with DI to tolerate polyuria and polydipsia for a prolonged period of time without water and electrolyte imbalance, patients with prolonged DI duration would eventually require desmopressin regardless of their tolerability to large amounts of drinking and UOP (3). This made the decision to prescribe desmopressin after discharge less interfered by the variations in the individual's tolerance and further explained the difference in the result of external validation between the prediction of a single dose of desmopressin and requiring desmopressin at discharge. Together, the external validation reinforced our finding that patients with higher peak Na levels and greater Na fluctuation had an increased likelihood of requiring desmopressin prescription at discharge.

Although a higher peak UOP predicted requiring at least a single dose of desmopressin in the internal validation (Cohort 2016–2019), it was not supported by the external validation (Cohort 2020–2021). The peak UOP prior to desmopressin could be influenced by multiple factors, such as perioperative fluid administration and the patient's tolerability to polyuria, which were almost impossible to standardize (1, 4). In terms of radiographic evaluations, previous studies have mentioned that tumors with suprasellar extension and tumors of greater cranio-caudal diameter were associated with increased risked of postoperative DI (2, 30–33). Our results showed that patients who require desmopressin had larger cranio-caudal tumor diameters. However, in our multivariate analysis, a larger cranio-caudal tumor did not predict the need of desmopressin. It is probable that the large variations in the cranio-caudal diameters may limit the predictive power, given that the interquartile ranges of cranio-caudal diameter in both groups were rather large.

External validations are necessary to assess the capability to generalize a prediction model on other similar populations (34). Although several predictive models were proposed in the literature to stratify patients following sellar surgery, external validations were not performed (3, 7–9, 30–32). In the present study, a second cohort from 2020–2021 was used to externally validate the potential predictors identified from multivariate analysis performed in the 2016–2019 cohort. Peak Na ≥ 150 and peak Na – minimum Na ≥ 10 in the perioperative period were validated externally as predictors of requiring desmopressin at hospital discharge. We proposed our postoperative management and discharge algorithm based on thehe two cut-off values. Specifically, the serum Na level in patients diagnosed with DI was monitored until at least POD 4, and any measurement showing peak Na ≥ 150 mmol/L or peak Na—minimum Na ≥ 10 in this period would render the patients as high risk. Although this finding may seem evident to experienced pituitary specialists at first, we found this to be of benefit in identifying patients at increased risk of prolonged DI and requiring desmopressin treatment after discharge. Based on this finding, we suggest a 1–2-day delay in hospital discharge in such patients to ensure complete resolution of clinical DI symptoms and to optimize the dosage of desmopressin required. In addition, prior to discharge, these patients should be warned of the higher likelihood of DI symptom occurrence and thoroughly educated about symptoms that indicate DI. Furthermore, this criterion is especially helpful to reinforce the safety when discharging patients with normal perioperative Na levels without desmopressin prescription. Patients presenting with polyuria without elevated serum Na level and increased fluctuation of perioperative Na are at significantly low risk of prolonged DI, and they could almost always be discharged without desmopressin. In this study, 129 patients from both cohorts met the criteria of peak Na <150 mmol/L and peak Na—minimum Na <10. Among the 129 patients, 128 were discharged without desmopressin.

This study was limited by its retrospective design. Additional limitations included the relatively small sample size, compared to large-scale multicenter studies. A relatively small sample size may limit the statistical power of multivariate logistic regression. Although combining the two cohorts can increase the sample size, external validation cannot be performed, and whether the identified predictors could be generalized to assist clinical practice would be unknown. It should be highlighted that both cohorts were reviewed retrospectively and the predictors identified in Cohort 2016–19 were not used to prospectively guide the management in Cohort 2020–21, and this could lower the bias of the external validation. Next, the 4-day postoperative monitoring period might be less available at centers where a fast-track discharge for pituitary surgeries is performed (35). However, our strategy could still be applied by arranging early postoperative clinical and laboratory follow-ups, and patients with large Na fluctuations detected should not be assigned to fast-track discharge.

Another limitation to the present study is the unavailability of copeptin measurements. The use of copeptin level to diagnose central DI has gained popularity recently (36, 37). Although one previous study reported a postoperative copeptin <2.5 pmol/L predicted postoperative DI with a positive predictive value of 81% (38), whether it is correlated to the need of desmopressin treatment remained unknown. Further, the quantification of copeptin level may be more time-consuming and less suitable in the immediate postoperative setting compared to the measurement of sodium levels (37). Still, copeptin level may be useful, especially in combination with our proposed criteria, to identify high-risk patients requiring desmopressin prescription after hospital discharge. Further studies are warranted to investigate the combined use of postoperative sodium and copeptin levels in the prediction of requiring desmopressin following sellar surgery.

## Conclusion

The clinical course and required treatment for patients with postoperative DI varied. Patients requiring desmopressin treatment were associated with longer hospitalization, higher UOP, greater perioperative Na fluctuation, and larger cranio-caudal tumor size. Elevated peak Na and greater peak Na-minimum Na values in the perioperative period were predictors of requiring desmopressin after hospital discharge. Patients with peak Na <150 mmol/L and peak Na—minimum Na <10 can be safely discharged without desmopressin.

## Data Availability Statement

The original contributions presented in the study are included in the article/supplementary material, further inquiries can be directed to the corresponding author/s.

## Ethics Statement

The studies involving human participants were reviewed and approved by Institutional Review Board of National Cheng Kung University Hospital (NCKU-IRB-Approval No. A-ER-110-147) Institutional Review Board of Taipei Veterans General Hospital (VGHTPE-IRB-Approval No. 2021-06-011AC). Written informed consent for participation was not required for this study in accordance with the national legislation and the institutional requirements.

## Author Contributions

J-SL and W-HW supervised and coordinated the study. C-EW and J-SL conceived and designed the study and wrote the manuscript. W-HW, M-YL, and J-SL performed the surgeries. C-EW, P-HL, C-CH, and P-FS obtained, analyzed, and interpreted the data. All authors revised the draft for important intellectual content and agreed upon the manuscript before submission.

## Conflict of Interest

The authors declare that the research was conducted in the absence of any commercial or financial relationships that could be construed as a potential conflict of interest.

## Publisher's Note

All claims expressed in this article are solely those of the authors and do not necessarily represent those of their affiliated organizations, or those of the publisher, the editors and the reviewers. Any product that may be evaluated in this article, or claim that may be made by its manufacturer, is not guaranteed or endorsed by the publisher.
